# Predicting Adverse Drug Reactions from Social Media Posts: Data Balance, Feature Selection and Deep Learning

**DOI:** 10.3390/healthcare10040618

**Published:** 2022-03-25

**Authors:** Jhih-Yuan Huang, Wei-Po Lee, King-Der Lee

**Affiliations:** 1 Department of Information Management, National Sun Yat-sen University, Kaohsiung 80424, Taiwan; abner0908@gmail.com; 2 Department of Healthcare Administration and Medical Informatics, Kaohsiung Medical University, Kaohsiung 80708, Taiwan; kdlee0704@gmail.com

**Keywords:** adverse drug reaction, social media monitoring, pharmacovigilance, machine learning, feature engineering, deep learning

## Abstract

Social forums offer a lot of new channels for collecting patients’ opinions to construct predictive models of adverse drug reactions (ADRs) for post-marketing surveillance. However, due to the characteristics of social posts, there are many challenges still to be solved when deriving such models, mainly including problems caused by data sparseness, data features with a high-dimensionality, and term diversity in data. To tackle these crucial issues related to identifying ADRs from social posts, we perform data analytics from the perspectives of data balance, feature selection, and feature learning. Meanwhile, we design a comprehensive experimental analysis to investigate the performance of different data processing techniques and data modeling methods. Most importantly, we present a deep learning-based approach that adopts the BERT (Bidirectional Encoder Representations from Transformers) model with a new batch-wise adaptive strategy to enhance the predictive performance. A series of experiments have been conducted to evaluate the machine learning methods with both manual and automated feature engineering processes. The results prove that with their own advantages both types of methods are effective in ADR prediction. In contrast to the traditional machine learning methods, our feature learning approach can automatically achieve the required task to save the manual effort for the large number of experiments.

## 1. Introduction

Drugs for medical purposes aim at saving one’s life and for improving the life quality. They are used to treat disease and to improve one’s physical fitness or mental status. However, drugs may cause unexpected effects or even adverse reactions on patients, called adverse drug reactions (ADRs). To prevent ADRs, clinical trials of drugs are conducted to analyze and evaluate the risks during the process of drug development. However, clinical tests are often very time-consuming and difficult to effectively detect all the possible adverse drug reactions due to the limitations on samples. A complementary way is to perform drug post-marketing surveillance by collecting patients’ opinions to construct data models to predict suspected ADRs. To tackle the crucial issues related to opinion modeling, in this work we perform data analytics and develop an efficient approach to automatically achieve the model construction procedure.

Traditionally, after drugs have been invented and released to the market, drug manufacturers, U.S. Food and Drug Administration (FDA) and the World Health Organization (WHO) continuously conduct pharmacovigilance to capture the uncovered ADRs as early as possible. The task of ADRs monitoring thus relies on the reporting systems taking feedbacks from relevant staffs and patients. However, the reporting system often needs a longer time to accumulate sufficient data to make decisions accordingly [1,2]. Moreover, these non-personal reports present some problems; for example, they are subject to errors in semantics or delayed in response time [3]. The reporting systems are far from being satisfactory. In contrast, with the increasing popularity of social media, more and more people are willing to share their medical experiences in social media [3,4,5]. Patients sharing their experiences on the social media often articulate medical issues and side effects previously not found or underestimated by clinicians. This channel thus offers a new way for ADR detection through extracting information from social media, including information of the side effects of drugs, drug usage compliance, the effectiveness of drug and relevant evidence. Many researchers are trying to develop new methods to identify and retrieve drug-related users’ opinions from social media, for example, [6,7,8]. Among the popular social media, Twitter that allows users to create short messages is considered as a rich source of user opinions on drugs. Though not all of the tweets posted by users are related to health issues, there are a sufficient number of tweets to augment the existing database as a source of adverse drug reaction information. It has been confirmed that the adverse drug events reported on Twitter were fairly consistent with the spontaneous reports [3].

Regarding the detection of adverse drug events in Twitter, there are several challenges to be solved. As already indicated by the relevant studies [9,10], the first issue is the sparseness of adverse drug events. As Twitter is a general social media with quite diverse content, most of the tweets are not related to adverse drug events. It results in a serious problem of data imbalance: the dataset has a skewed class distribution of ADRs and non-ADRs. The second issue is to do with data features with a high-dimensionality. This is mainly caused by the unstructured texts of tweet posts. The quantity of features extracted from the posts can be prodigious, so it is difficult and laborious to select useful features and develop suitable encode schemes to represent data for predictive modeling. The third issue is regarding the diversity of the tweet terms. As anyone can use their own terms based on personal feeling to create a tweet to express a subjective opinion, even in describing his situation on the same ADR. Diversity of the tweet’s sentence causes the natural language processing (NLP) to be more difficult but machine learning methods are able to solve this problem.

Our study aims to tackle all these challenges and contains several features. We first perform a comprehensive experimental analysis to evaluate the major procedures of traditional machine learning methods for ADRs detection from different perspectives, including data balance, feature extraction and selection, and ensemble learning. Then, we develop a deep learning-based approach that adopts the state-of-the-art BERT model [11,12] with a new adaptive strategy to alleviate the problems often occurring in traditional methods. A series of experiments have been conducted to evaluate the performance of machine learning methods with a manual (i.e., traditional methods) and an automated (i.e., deep learning methods) feature engineering processes. Finally, we have analyzed and compared the effectiveness and efficiency of both types of methods. Compared to traditional methods, our method with feature learning can save a lot of manual efforts in orchestrating the large number of experiments and provide a more efficient solution in ADR prediction.

## 2. Related Work

Nowadays patients’ tweets mentioning the drugs and the related side effects provide most popular information source for post-marketing drug safety surveillance. There are two types of computation methods in use for building tweet-based classifiers at present: feature engineering-based methods and feature learning-based methods. The former often involves two operating stages: employing NLP techniques for extracting data features from the tweets, and then performing traditional machine learning methods for building models for ADR classification. The first stage deals with the unstructured tweets and uses natural language processing techniques at different text (lexical or sentence) levels. Specific characteristics are analyzed, such as corpus construction and part-of-speech (POS) analysis, medical entity identification, and drug adverse event (ADE) relationship extraction. At this stage, various types of data features are defined and extracted, mainly manually, from the texts to represent the data (tweet). With these features, the second stage then constructs classifiers accordingly by different computational methods.

In former studies on text classification, traditional machine learning algorithms were often used, such as Naive Bayesian Classifier (NB), Decision Tree, Support Vector Machine (SVM) and Logistic Regression (LR), to provide reasonably good performance. For example, Chee et al. used a bootstrapping method to increase positive samples to balance the data distribution, and they developed an ensemble model to identify ADR by combining the SVM and NB classifiers [13]. Additionally, Plachouras et al. implemented a SVM based classifier to categorize tweets [14]. Sampathkumar et al. utilized Hidden Markov Model (HMM) to recognize if a post of the healthcare forum has provided any drug side effect information and to extract the targeted content [15]. Nikfarjam et al. adopted different types of supervised machine learning algorithms, including SVB, NB, and Maximum Entropy (ME), to predict whether ADRs existed in a social media text [16]. Patki et al. deployed a two-step approach for ADR detection. They first used Multinomial NB and SVM to classify reviews into ADR or non-ADR, and then performed text analysis to identify potentially dangerous drugs from the ADRs related mentions [17]. In addition, some studies proposed to infer the associations between the side effects of approved drugs to further predict potential side effects for new drugs, for example [18,19].

To enhance the performance delivered by a single comprehensive classifier, researchers proposed to ensemble multiple models or algorithms. Intuitively, they combined multiple machine learning models in order to keep the advantages and remove the disadvantages of the individual classifiers. In data modeling, each type of model can provide specific views to interpret the original data. Based on this observation, some ensemble learning approaches used the same machine learning algorithm to build multiple models while each model was trained by different subsets or variables of the data (such as RandomForest or Adaboost). There were also works using the same dataset to train models by different machine learning methods, and then taking the voting or average results as the final decision. For example, Liu et al. used NLP techniques to transform texts collected from health-related forums and Twitter into the lexical, syntactic, and semantic features. They then trained ensemble learners, selected *k*-best models, and made the final decision by fusing strategies to predict ADEs [4]. Wunnava et al. adopted heterogeneous classification algorithms (including SVM, DT, and LR) and combined them as an ensemble learner to extract ADRs narratives to form the FDA adverse event reports [20]. They have found that the stacking-based ensemble combiner method can outperform the simple majority-voting method. Recently, Kim et al. used a stacked ensemble method with a search-based structured prediction algorithm to recognize ADEs from electronic health records [21]. Moreover, Liu et al. developed a semi-supervised ensemble learning framework to augment the training data and improve the skill of the diverse base classifiers [7]. They used the ensemble classifier to label the unseen data and re-train the base classifiers, and the workflow continued until performance of the ensemble classifier was stable. Their work can obtain better results than the traditional training steps using single classifier only. In addition, El-allaly et al. proposed a multi-task transfer learning-based approach to extract ADEs from clinical textual data [22]. They converted the ADEs extraction task into a dual-task of source mention extraction and attribute–relation extraction, and then used the developed approach to solve the two tasks. 

Now using deep neural networks is a breakthrough for classification tasks. The deep networks can achieve superior performance in many applications, especially in computer vision and natural language processing. Particularly in the applications involving with semantic cognition (such as machine translation, sentiment analysis, and chatting), traditional machine learning and features engineering methods are not able to capture the meaning of spoken sentences. For such applications, deep learning NLP techniques outperform traditional machine learning methods. Therefore, to exploit the optimization power of deep learning, researchers have applied this method to detect ADRs from unstructured, manually generated social media texts [23,24]. A primary study in using deep learning for pharmacovigilance was the work by Cocos et al. [25]. The authors utilized a bidirectional LSTM trained on a sample Twitter dataset to identify drug side effects. There were also some studies applying the deep learning-based methods to biomedical literatures for ADRs detection [26,27]. For example, Basaldella and Collier proposed an embeddings approach to improve the deep learning algorithm performance and applied their system to the forum Reddit, particularly with a focus on the bio-subreddit [28].

Among others, the pre-trained language model BERT has shown its superior performance in language-related applications. It used an extremely huge corpus to train a language representation model from a deep learning architecture designed by Transformers [11]. The capability of a pre-trained model depends on the quantity of training data, and it is difficult to obtain sufficiently large amount of data and computing power to well-train such a model. Therefore, many biomedical and pharmacovigilance studies have adopted BERT as a language representation component to improve the corresponding NLP tasks [29,30,31]. For example, Fan et al. recently proposed a deep learning-based approach that adopted Bidirectional Encoder Representations from BERT-based models for ADE detection and extraction [31]. They used a manually labelled dataset containing a large number of reviews from the health social media forum WebMD and Drugs.com to evaluate the model. As a result, this model obtained the best results in their task. It has the potential to be applied to other healthcare and information extraction tasks. Following this trend, in this work we develop a new approach based on BERT and conduct experiments for performance comparison.

## 3. Data Description

In this study, we used the dataset annotated by Sarker et al., in which each tweet has an identifier and an ADR label [32]. We used this dataset because it is a real dataset publicly available, and most importantly, it is very imbalanced (different from those datasets used in other studies for pharmacovigilance research, for example DailyStrength [6], WebMD [31], ADE corpus [24]). The tweets were written in English and the data were collected in April, 2017. The methodology presented in this work is not specifically designed for this dataset; it is applicable to others. As some tweets in this dataset have been removed for some reasons (e.g., deleted by the original users), the data amount used in this study is slightly less than the original one used in the literature. It contains 6842 data records: 737 ADRs and 6105 non-ADRs. Obviously, it is an extremely imbalanced dataset and tends to cause the imbalanced class distribution problem, and thus to obtain results of high accuracy (biased toward the non-ADR class) but low f-score. Therefore, for this type of dataset, it is important to train a predictor to improve the f-score without the loss of accuracy. In this work, the experiments and evaluations are conducted based on this popular dataset. However, the limitation on the data source should be noted, such as that the completeness of this source may be not sufficient, and there could be a potential bias in the selection of social posts.

Table 1 lists the details of the dataset, which includes nine categories of data features with 8496 different features in total. The feature categories are “text” (word terms), “synset” (synonyms of verbs, nouns and adjectives), “sentiment” (positive or negative of the user opinions), “cluster” (clusters of the texts), “structural” (structural properties of the texts such as the number of words, sentences in the texts), “adrlexicon” (whether a tweet contains a ADR lexicon), “topic vector” (topic keywords), “topic” (the correlation score for the keywords included in each cluster) and “goodbad” (whether a sentiment change is detected in a tweet). In this study, we use the feature extraction program provided by [16] to extract data features with different encoding formats from the text content. The feature names and the numbers of feature dimension are also listed in Table 1. It is helpful to capture the as much ADR information as possible, through combining all features into one data record. For this dataset, the feature extraction process generates 8496 features in total. However, the number of dimensions is much larger than the amount of data, 6842, and this raises the curse of dimensionality [33]. It is therefore very important to select critical features for building classifiers. In the experiments, we investigate serval approaches to overcome the problem of class imbalance and to improve the effect of high feature dimension for better performance.

## 4. Research Method: Data Balance, Feature Selection, and Deep Learning

As mentioned above, posts collected from social media provide useful information about the patients’ opinions. To identify ADRs, in this section we describe how we perform data analytics on these social media posts from two aspects to tackle the related issues and to enhance the predictive performance. The first is to consider critical factors related to data processing for feature engineering, including the traditional data balance and feature selection; and the second, to develop more effective models and methods for feature learning (such as deep learning-based methods). Further details are given below.

### 4.1. Tackling the Data Imbalance Problem by Resampling and Ensemble Learning

The class distribution of ADRs and non-ADRs could present a very severe between-class imbalance problem. To obtain a higher accuracy, a model by machine learning often biases toward the non-ADR class with a relatively large number of data samples. Thus, an additional data balancing procedure is required to conciliate this problem. Before developing more advanced computational methods for classification, we investigate the effect of data balance by two types of methods (i.e., data resampling and ensemble learning) described below to derive models from data with imbalance class distributions.

The first type is to adopt direct-resampling techniques. Intuitively, re-balancing the class distribution often involves over-sampling or under-sampling techniques. The simple over-sampling technique duplicates the minority class instances to equalize the data amount of different classes. However, adding data this way may not improve performance because it does not provide additional information to the learning method about how to identify minority class instances. Therefore, most over-sampling techniques attempt to analyze data properties to synthesize more meaningful and informative data for the minority class. Another technique is under-sampling, in which class balance is performed by decreasing the amount of majority class data. A simple technique is to randomly sample data from the majority class with the same amount to the minority class. However, this approach also has drawbacks: it might sample data with similar representations in the feature space, leading to the majority class lost many useful data to support the original representation. In this work, we use the imbalanced-learn python package [34] to perform the re-sampling process. This package provides many re-sampling methods reported by literature works, and we design a series of experimental evaluations to examine if resampling methods can be used to improve the predictive performance. Three steps are performed: at first, we split the data into training and test datasets; then we apply the resampling techniques on the training dataset only in order to re-balance the skewed classes and keep the test dataset in original distribution; and finally, we use the rebalanced dataset to train the classification algorithms.

In addition to the above way that separates the steps of data quantity processing and model learning, a popular alternative to alleviate the class imbalance problem is to adopt ensemble learning that couples data modeling and data balance in the same procedure. This type of learning takes a multi-view to investigate the dataset, and many operating strategies (such as bagging, boosting, and stacking) can be employed to work with the learning method chosen (such as decision tree). Some strategies sample the subsets from the original dataset and then use data of different subsets to train models or the same dataset on different learning algorithms to build the overall model; others consider different dimensions of dataset to train individual models. All these approaches attempt to inspect data from multiple angles, though they are different in operating details. With such a specific property, ensemble learning methods are more capable to overcome the effects caused by imbalanced classes. Moreover, ensemble learning methods are easy to combine with resampling and cost-sensitive techniques to provide more effective prediction.

### 4.2. Solving the High-Dimension Problem by Feature Selection

Another issue needs to be seriously considered in Twitter posts is the feature dimension. The dataset collected from a social forum is often composed of a lot of free-style messages. It means that the data (i.e., the tweets) includes a textual document in non-structured format. We also have to format the text for classification algorithms to understand and process. Moreover, the words with ADR meanings are obscure, implicit, and not easy to detect. Sometimes an ADR mention is not just a single word, but a set of words, or even a sentence. It needs to be recognized through context analysis. As we do not know in advance what kinds of word relations or types the ADRs are built on, we thus apply different kinds of feature extraction methods to the same sentence and the results are concatenated to be one data record. As a result, a short sentence in the Twitter post is often expanded to form high-dimensional data.

For the dataset used in this work, the number of features is much larger than the amount of data. Though rich data features are considered helpful for improving the model performance, the functional roles of data categories are to be examined carefully to verify if they are beneficial to the model or not. One way to solve this problem is to adopt feature selection methods to choose a subset of the original features to maximize the modeling performance of a learning algorithm. Consequently, the dimension of feature vectors can be reduced so as to reduce the overfitting situation and the computational effort in learning a model. 

Traditionally, the positive effect of data features is the major aim of a classification task; it is also important for presenting the negative effect. To inspect the negative effect of each feature category listed in Table 1, former studies have employed the leave-one-out method to remove individual category, and observed how the predictive performance (i.e., the metrics of accuracy and f-score) changed. Their results show that though most of the features are helpful, the performance changes are not obvious. To amend it, here we further investigate these effects by examining and analyzing the impact of each feature category in detail. A series of experiments are conducted to identify whether the rich feature categories contribute to data modeling effectively, and whether each category can be substituted by others in the modeling procedure to maintain the same level of performance. If the most appropriate feature combination can be used, it is expected that the efficiency and efficiency of the models can be improved. In our application, we aim at the metrics of accuracy, f-score, and AUC (area under the Receiver Operating Characteristic curve).

One of the best ways to select features is the type of filtering-based methods that employ statistical techniques to score features and determine what to be reserved accordingly. A popular filtering method is the univariate filter which evaluates each feature one by one independently. As the calculation for the Univariate filter is fast and the predictive performance is generally acceptable, therefore, we adopt this method for feature selection. Univariate selection performs statistical test for non-negative features to select *k* best features. That is, it uses common univariate statistical test for each feature, measures the relationship between the feature and the strain, and selects the best features accordingly. This work uses the simple and efficient tools scikit-learn [35], with the three criteria it provides for feature evaluation: chi-square, Pearson correlation, and ANOVA F-value. More details about the three algorithms are given in [36], and the evaluation results are presented in the experimental section.

### 4.3. Enhanced Deep Learning for ADR Recognition

The above methods for class balance and feature selection have pipeline workflows so they are not able to guarantee the best overall performance through pursuing the best results individually at each stage and then combining them together. Therefore, we press on to integrate the above stages and build an end-to-end approach via the deep neural model. The model is designed to capture the long-term dependencies of the input sequential texts (tweets). It can combine both functions of feature extraction and feature selection, via temporal contextual information. Working in this way, this model is able to detect the key words as well as the context information just as humans can. This is a crucial characteristic for our application here because the ADR symptoms are often represented by various types of sentences and require human-like comprehension for precise recognition.

To use a deep learning approach to achieve a language-based task, it is difficult to deal with the diversity of words, especially on the small corpus dataset, because there are not sufficient data to build up a model through learning the hidden patterns from the small corpus. Adopting the transfer learning techniques, we can choose a pre-trained model (trained by a large-scale corpus on a particular application domain) and then use a specific dataset to re-train this model to derive a fine-tuned model. In this way, regardless of the relatively limited data in the specified domain dataset, the re-trained model can still achieve outstanding performance.

**Figure 1:**
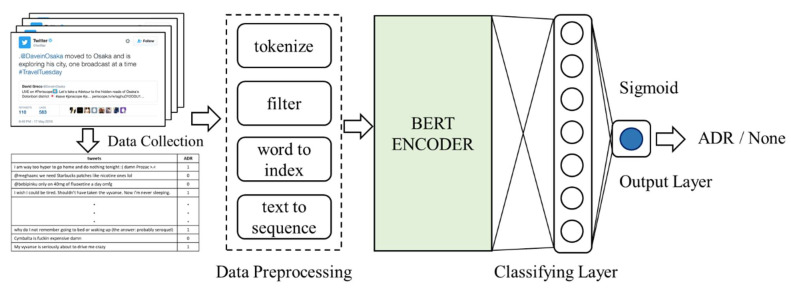
The proposed network architecture.

In this study, we adopt BERT as the encoder to develop our approach. BERT, the deep learning technique based on the transformer network architecture, has been pre-trained on a large-scale corpus. The end part of the proposed model is composed with a classifying layer. It can be a simple fully connected layer or a more complicated RNN-like layer. The output layer uses the sigmoid activation function to generate a prediction with a real number between the range of 0 and 1. Here, an output number over 0.5 means that the model will take this observation as an ADR. Figure 1 illustrates the deep neural networks-based architecture of our ADRs classifier. As already shown, before the tweets are sent into the model, each of them is parsed by a data preprocessing procedure, including steps of stop words elimination, html tags elimination, URL links elimination, and tokenization. A vocabulary dictionary is conducted with all corpus terms and each word is turned into a number encoded by the index of the corresponding term in the dictionary. We align all input sentences to sequences of length 32 (the average data length); the over-length sentences are truncated while short sentences padded with zero values. In this network model we use a fully connected layer with size of 768 neurons as the classifying layer.

In addition to the network architecture, it is important to enhance the training method to reduce the serious imbalanced class effect in ADR detection. As the deep neural network is trained by the stochastic gradient descent optimization algorithm, it requires the choice of loss function to repeatedly estimate the loss of the model and then to update the network weights accordingly. The loss function represents the primary training objective for a neural network, and the training performance largely depends on the choice of the loss function. Different loss functions have been used for the training deep neural model. Among others, the cross-entropy function has been widely adopted because it couples with a commonly used Softmax output layer to undo the exponentialized outputs. Meanwhile, its properties are closely related to the Kullback–Leibner (KL) divergence that describes the differences between two probability distributions [37]. Consequently, minimizing the cross-entropy function is similar to minimize the KL divergence. However, this function is not implicitly flexible about the amount of information to be back-propagated. Therefore, instead of using a static loss function, we propose a new objective function based on the method of batch-wise self-adaptive weighting. With the adaptive loss function, the model becomes flexible in estimating its error to dynamically capture the characteristics of the data and the learning environment. The model can thus be forced to learn only the most discriminative and contributive features. As the model can capture the inherent trade-offs between the classification accuracy and the robustness to noise, the trained model can thus be more immune to the overfitting problem. 

Our objective function enables the deep neural networks model to balance the class weights at each batch step in the training stage. With an adaptive function to dynamically guide the learning, we can alleviate the data imbalance problem to improve the performance of the model obtained. The following set of equations (i.e., Equations (1)–(6)) quantitatively explain the proposed method. Equation (1) presents the cost function, which indicates that given a loss function *L*, it is able to compute the distance between the prediction $\hat{y}$ (by the model) and the ground truth y based on the data distribution, in which θ represents the set of trainable parameters of the deep model. The goal is to approximate the distribution of model mapping based on the input x and the prediction $\hat{y}$, so it will be as close as possible to the empirical distribution. It means that the cost needs to be minimized, and we can thus turn Equation (1) into an optimization problem as described in Equation (2). Here, the deep model adjusts the trainable parameters (i.e., θ) to enable the probability distribution of the model’s output to reach the maximum log-likelihood with the training data {$x_{1,2,\ldots ,n},\;y_{1,2,\ldots ,n}$}. With Equation (2) into Equation (1), we can revise Equation (1) to obtain Equation (3). In such a gradient method, the optimization algorithm can update the parameters based on the training set, as described in Equation (4).

(1) $$\begin{array}{cc} J\left(\theta \right)= & E_{\left(x,y\right)\sim {\hat{p}}_{data}}L\left(\hat{y},\;y\right)\\ \hat{y}= & model\left(x;\;\theta \right) \end{array}$$

(2) $$\theta_{ML}=\underset{\theta }{\arg\;\max}\;\overset{n}{\underset{i}{\sum }}\log\;p_{model}\left(x_{i},\;y_{i};\theta \right)$$

(3) $$J\left(\theta \right)=E_{\left(x,y\right)\sim {\hat{p}}_{data}}\;\log\;p_{model}\left(x,\;y;\theta \right)$$

(4) $$\nabla_{\theta }J\left(\theta \right)=E_{\left(x,y\right)\sim {\hat{p}}_{data}}\;\nabla_{\theta }\log\;p_{model}\left(x,\;y;\theta \right)$$

As can be observed in Equation (1), the type of loss function influences the deep model considering the learning patterns from data. For an imbalanced dataset, if the traditional cross-entropy is used as the objective function, the model tends to take the majority class (i.e., non-ADRs here) as the prediction result, because the majority class provides the minimum loss for the training data. However, this results in a serious bias. It is necessary to amplify the error when the instance is an ADR and the model gives a wrong answer. Therefore, as Equation (5) shows it, the binary cross-entropy loss function is multiplied by a weight *w_i_* to reduce the class imbalance effect. In this equation, $x=\left\{x_{1},x_{2},\ldots ,x_{n}\right\}$, $y=\left\{y_{1},y_{2},\ldots ,y_{n}\right\}$, and n is the data size. In contrast to the kind of studies with fixed weights used to balance the skewed classes, our weight here is computed for each mini-batch, depending on the class distribution of the batch data. The proposed batch-wise adaptive weight formula is shown in Equation (6), in which B is the batch size, y∈{0, 1} (an ADR datum has a *y* value of 1), and the term ($1/B)*\overset{B}{\underset{i=1}{\sum }}y_{i}$ means the percentage of the ADR class in the batch data. For example, assuming that ADRs are the minority in the mini-batch (the same as in the training set) and they occupy 10% of the mini-batch, we can calculate ($1/B)*\sum_{i=1}^{B}y_{i}=0.1$ and then obtain ${\left(-1\right)}^{y_{i}}={\left(-1\right)}^{1}=-1$. Thus, we can derive $w_{i}=1-0.1=0.9$. Meanwhile, for an instance of non-ADR data, $y_{i}=0$, we can derive $w_{i}=0+1*\left(0.1\right)=0.1$. Through the above steps, during the procedure of learning, the weights can be dynamically adjusted according to the class distribution of the mini-batch, so more penalties are given to examples of ADR data wrongly predicted. This way, our method can more precisely describe the relation between the model and the data in practice, and it is thus not necessary to take special care on the data imbalance problem.

(5) $$Loss\left(x_{i}\right)=-w_{i}\left[y_{i}\log\left({\hat{y}}_{i}\right)+\left(1-y_{i}\right)\log\left(1-{\hat{y}}_{i}\right)\right]$$

(6) $$w_{i}=y_{i}+{\left(-1\right)}^{y_{i}}\frac{\sum_{i=1}^{B}y_{i}}{B}$$

## 5. Experiments and Results

In this section, we present a series of experiments conducted in three phases for performance evaluation from different perspectives. The first and the second phases were to inspect the performance of traditional feature engineering methods. In the first phase, we investigated the effect of data balance and then assessed the performance of ensemble learning methods. In the second phase, we examined the effect of feature selection in data modeling. Finally, we evaluated the proposed deep learning approach that has the special characteristics of automated feature selection and learning. 

### 5.1. Results of Data Balance by Resampling Techniques

In this phase, we first adopted the method presented in [16] to obtain the baseline performance. As some data used in the baseline work were no longer available at the time of experiments, we cannot directly take their results for comparison. We thus replicated their experiments by using the currently available 6842 posts with 737 ADRs. To ensure the identical experimental conditions for an objective evaluation, we used the text preprocessing and feature extraction programs provided by the original work. The same data preprocessing procedure and the same SVM classifier were adopted. In our implementation, we used the grid search technique to derive the optimal parameters to tackle the class imbalance problem and to maximize the performance of the original method (i.e., SVM). To examine the performance, we also adopted logistic regression (LR) to test this dataset; in fact, many other relevant studies have considered it effective for binary classification. Table 2 shows the re-run results that are similar to the original work. It means that the dataset we downloaded has a data distribution quite similar to the original one. In the following experiments the results obtained here are used to be the baselines for comparison.

To evaluate the effect of data resampling (i.e., under-sampling and over-sampling), we conducted two sets of experiments. The results of using under-sampling technique with different popular classifiers are presented in Table 3. As shown, we first tested logistic regression without any balance technique. Compared to the results in Table 2, the LR model without any balance method shows higher accuracy but lower recall and f-score. It means that the model preferred to predict data to be non-ADR biasedly as described previously. Next, we evaluated the random under-sampling method that randomly took the same amount of data from two classes. It was a down-sampling process, which resulted in the situation that there was not sufficient amount of data to support the model to recognize the relations between observations and targets. Therefore, the random under-sampling method produced a model with a better recall but losing accuracy, precision, and f-score. It means that the model tended to predict non-ADR data to be ADRs. The results of other classifiers with the resampling technique are also listed in the table. Overall, as can be observed that the methods based on the neighborhood cleaning rule [38] or the edited nearest neighbor [39,40] can obtain better performance. This is because they employed different kinds of strategies to remove neighborhood redundant or overlapping data.

Another set of experiments have also been conducted to evaluate the effect of over-sampling technique for comparison, and the results are presented in Table 4. As can be observed, compared to LR without data balance, even the random over-sampling technique is helpful for the classifier to improve the f-score. Next, we tested the SMOTE-base up-sampling algorithms that used certain kind of strategy to synthesize the data of minority class [36,45,46,47]. As shown in the table, these methods have consistent and similar results. Although these methods were able to improve the f-score, they did not outperform the random over-sampling technique. Finally, a hybrid method was employed, in which the over-sampling technique was followed by the under-sampling method [48,49]. They are still considered up-sampling techniques. Synthetic up-sampling was first performed in the minority class and then the down-sampling technique was used to clean the redundant data. For this dataset, any kind of over-sampling technique was able to help classifiers to resist the imbalanced class effect and all classifiers can obtain a better f-score.

### 5.2. Evaluation of Ensemble Learning Methods

As indicated above, ensemble learning provides an alternative way to alleviate the problem of imbalanced data classes. With the underlying principle of looking into data from different perspectives, in general the ensemble learning methods have better abilities to overcome the effect of imbalanced classes [4,7,20,22]. To evaluate the performance of ensemble learning, in this set of experiments, we tested several popular methods reported in the literatures with superior performance and compared the results. These methods included decision tree (DT) with data balance and bagging technique [50], Random Forrest (RF) with data balance [51], EasyEnsemble [52], and RUSBoost [53]. The results are presented in Table 5.

As shown, DT with data balance bagging technique has higher accuracy and f-score but a lower recall than the other three methods; RF with data balance, EasyEnsemble, and RUSBoost have similar performance in the metrics of recall, f-score, and AUC. Judging from the more objective performance metric f-score, here the ensemble methods did not outperform those presented in the above section (i.e., results in Table 2, Table 3 and Table 4).

### 5.3. Results of Classification with Feature Selection

In the second phase, we performed a set of feature profiling trials in which different numbers of features were selected, to examine the details of the feature selection process during data modeling. Based on the results shown in Table 2, here we chose the logical regression method for further evaluation, and used the optimized parameters obtained above, to solve the problem of imbalanced classes. In the selection of features, we measured the chi-square, Pearson correlation, and ANOVA F-value to obtain the scores between each pair of feature and class. Then, the scores were sorted and the features with the *k* highest scores were selected accordingly.

**Figure 2:**
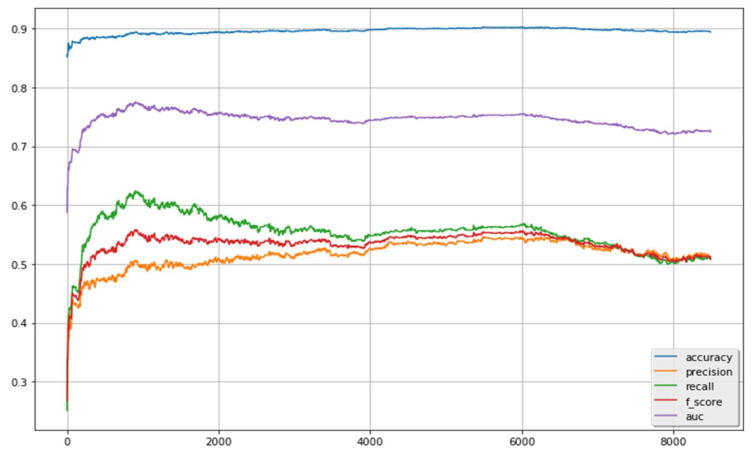
Results of the classifier with feature selection scheme.

Figure 2 illustrates the results for selecting different numbers of features (indicated in the *x*-axis). As demonstrated, when a small number of features were selected, the predictive performance cannot reach to a reasonably good level, because no sufficient information was extracted to support the classifier to correctly identify the labels between ADRs and non-ADRs. Following the increase of the selected features, the performance was gradually improved. When the number of selected features reached 1000, the best performance could be obtained. After this, there was no obvious improvement when the number of features selected was increased to a certain extent. The performance notably declined when more and more features were selected and approximated to the situation without feature selection. It means that not all features can provide positive information to the classifier. Some features may give noise signals or even negative information. Such features may obstruct with each other and thus damage the predictive performance. The feature selection techniques can remove such useless features and help the classifier focus on the important ones.

Table 6 lists the performance of the LR classifier coupled with both feature selection (FS) and data balance (DB) techniques (the second row). Compared to the classifier with only class balance technique (the first row), the feature selection scheme can better enhance the efficiency and effectiveness of the classifier. One more set of experimental trials has been conducted to further verify the importance of the features determined by the feature selection scheme. Here, the best 1000 features chosen by the feature selection scheme were removed (i.e., not allowed to be selected), and only the remaining features were used for data modeling. The third row of Table 6 shows that the performance significantly declined, and the importance of those features selected is duly confirmed.

To examine which categories of features were relatively important and thus selected, we summarized the numbers (Selected and Original columns in the table) and ratios (Category and Overall columns) of selected features belonging to different categories in Table 7. It shows that features of “text”, “synset”, and “cluster” categories were favored, and the numbers (and ratios) of “structural” and “goodbad” features were obviously smaller than others. In addition, it can be noted that the category of ADRlexicon has a highest selected rate (i.e., 100%). The reason could be that there were only two features in this category and they were related to the ADR words. In contrast, the most frequently selected category was synset (with 471 features selected). The reason could be that this category included a large number of meaningful text features (i.e., 2000 features) filtered by the text processing techniques and it thus has a higher probability to be selected.

### 5.4. Evaluation of Deep Learning Methods

To verify the effectiveness of feature learning, we conducted a series of experiments to compare different deep learning methods. We evaluated the results of BERT pre-trained model with different objective functions, including the self-adaptive function we designed in this work. Though some other studies have employed deep learning-based approaches to develop models for ADR prediction, they were not directly compared with ours for they mostly provided extra domain information to the models. Alternatively, to perform an objective evaluation, we thus chose the most relevant work ([54]) that used the same dataset for deep model development. We downloaded the programs to replicate the experiments so as to ensure that the experiments performed with the same test environments and conditions. The results were evaluated by the cross-validation method. In the experiments, we used the built-in Stratified K-Folds cross-validator of the scikit-learn package with K = 5, because this technique can ensure the same ratio of class distribution of the training and test sets as the original dataset.

Table 8 lists the experimental results (based on ten independent trials), showing the accuracy, precision, recall, f-score, and AUC. The first three sets of results were obtained from the trials with different deep models described in [54], including CNN (convolutional neural network), CRNN (convolutional recurrent neural network), and RCNN (recurrent convolutional neural network). Meanwhile, this table presents the results produced by various BERT-based methods (i.e., the same architecture with different objective functions). The objective functions include the binary cross-entropy (BCE), the mean square error (MSE), and our proposed approach batch-wise adaptive weighting (BAW). These results show that using the BERT pre-trained architecture can obtain improvement in major metrics (2~6% in accuracy, 1~10% in f-score, and 11~18% in AUC), if compared to other models based on deep learning techniques (i.e., CNN, CRNN, and RCNN). Among others, the proposed BAW method has relatively better performance. To sum up, BAW result has a higher f-score means that the better balance tradeoff between recall and precession can be obtained; and a higher AUC shows that the prediction abilities of the model are consistent at different levels of thresholds. 

To contrast the above method of adaptive weights, we further evaluated the binary cross-entropy function with a loss of fixed weights for the classes of non-ADR and ADR. Two strategies were used: weights of 0.1 and 0.9 (BERT with fixed weights-1 in Table 8) and weights of 0.2 and 0.8 (BERT with fixed weights-2) for non-ADR and ADR, respectively. The results show the latter strategy is better than the former, and our batch-wise adaptive weighting method can outperform. It indicates that the penalty weight is a critical factor in model training. If the weights are not chosen carefully, not fully following the class distribution or wrongly assigned, they can damage the prediction ability of the model. In the proposed approach, the weights can self-adapt to the training data. Consequently, developers do not need to determine the weights manually. A strategy with carefully pre-fixed weights can consider the class distribution with an overall view, but it can also cause the over-penalty or under-penalty of the loss computation at different batch scales. To tackle this disadvantage, our proposed strategy has encountered the data dynamics in the training procedure; it can adapt to a dynamic training environment to reduce this uncertainty and achieve the best results.

### 5.5. Discussion

In the above sections, we implemented different types of traditional methods and applied them to address the issues related to ADRs classification from a variety of perspectives, including data distribution, feature selection, and feature learning. The resampling techniques showed that the under-sampling methods based on the family of neighborhood cleaning rule algorithm can enhance the model performance up to 5~6% in the metrics of f-score and AUC; in contrast, the over-sampling techniques were relatively less effective that can improve the results up to 3–4% in both f-score and AUC. Though many studies showed that the ensemble learning method could be applicable to the skewed dataset to reduce the imbalance effect, however, for the dataset used in this work, the ensemble algorithms did not achieve the same performance level as the resampling techniques did. It is because of the curse of dimensionality caused by the high feature dimension. Under such circumstances, reducing the number of features by a careful feature selection should provide a solution to improve the classification performance, and the experimental results supported this method. Though the results can be improved, the feature selection scheme has the disadvantage of heavy computation required to score features and determine the suitable number of features. As explained, the abovementioned approaches have to handle a lot of processes in data preprocessing and feature engineering and these processes are often arranged in a pipe line procedure. Working in this way, the decisions made at any stage greatly influence the final performance. Therefore, the model developers have to carefully organize the relevant details at each stage. In practice, it is not easy to find which stage is causing the unexpected results and the developers thus have to laboriously perform and differentiate the experiments for improvement. 

To save the human users’ effort, we have adopted and evaluated deep learning methods to identify ADRs in tweets. With special characteristics, they can perform self-justification in data encoding and automated feature engineering. There is no need for the developers to consider the modeling details on the training and developing stages. In this study, the BERT-design for language encoding is used to tackle the diversity problem of ADRs-related terms, and the pre-trained model is then applied to deal with the imbalanced dataset. Moreover, a batch-wise adaptive weighting objective function is also designed to dynamically balance the skewed classes distribution during the model training. As indicated above, such an adaptive function can capture the inherent trade-offs between classification accuracy and robustness to noise. As a result, our objective function used in training can provide useful guidance in the search of appropriate network parameters; it has important impact in performance. The evaluation results have confirmed its effectiveness: comparing to other deep learning methods, our approach is able to obtain the best overall performance; in other words, our method not only has good performance on the imbalance dataset but also the confidence on the correct predictions. Such advantages are mainly brought about by the automatically discovered regularizations, so the model could have robust results for unseen data.

## 6. Conclusions

In this work, we emphasized the importance of post-market surveillance and took the social posts as the patients’ opinions to construct predictive models for ADR detection. To tackle the critical issues related to ADR predictions, we performed data analytics to identify ADRs from the viewpoints of data balance, feature selection, and feature learning. The methods for data balance and feature selection were integrated to ensemble learning for classification to consider both data and modeling together. We then designed more sets of experiments to investigate the performance of different data processing techniques and data modeling methods. From these experiments, we got the chance to observe the methods of effective classifiers. 

After the above analytical review of different methods, we further evaluated the deep learning-based methods able to perform feature learning to improve the efficiency in building classification models. Most importantly, we proposed a new objective function based on the method of batch-wise self-adaptive weighting. With the adaptive loss function, the model becomes flexible able to estimate its error to capture the characteristics of the data and the learning environment. In this way, the data imbalance effect can be alleviated and the model obtained is more immune to the problem of overfitting. To verify the proposed approach, we conducted a set of experiments to compare it with other deep learning-based methods. The results show the effectiveness and efficiency of our approach in ADRs detection. Overall, both traditional and deep learning methods are effective in prediction. The traditional machine learning with a carefully performed feature engineering procedure could obtain similar performance in f-score to our deep learning method. Nevertheless, our method with feature learning can save much manual efforts in orchestrating the large number of experiments. To model developers, it is preferred to provide a more efficient solution in ADR prediction. 

The proposed method shows prospects for further research. First, the restrictions of the datasets can be overcome. We are now collecting more posts from relevant social forums to enrich the dataset to further evaluate the presented data analytics system. Second, we are investigating other advanced techniques to improve the model performance, such as performing data clustering and developing new learning functions that can more aptly characterize the ADR data to guide the training process. Finally, we plan to train a cross-linguist model that is able to analyze social messages written in different languages.

## Figures and Tables

**Table 1:** The feature names extracted from the dataset.

Feature Name	Dim	Description
text	5000	*N*-grams, *N* = 1~3
synset vector	2000	the tf.idf measure for each derived synonym
cluster vector	981	cluster terms tf
topic vector	500	the topic terms that appear in the instance
sentiments	5	the sum of all the individual term-POS (part-of-speech) sentiment scores divided by the length of the sentence in words
good/bad	4	four features: MORE-GOOD, MORE-BAD, LESS-GOOD, and LESS-BAD
structural features	3	length: lengths of the text segments in words presence of comparatives and superlatives: these are binary features and these items are identified from the Stanford parses of the text segments presence of modals
ADRs lexicon	2	The first feature is a binary feature indicating the presence/absence of ADR mentions. The second feature is a numeric feature computed by counting the number of ADR mentions in a text segment and dividing it by the number of words in the text segment.
topics	1	sums of all the relevance scores of the terms in each instance

**Table 2:** The baseline results by SVM and logistic regression.

Methods	Accuracy	Precision	Recall	F-Score	AUC
SVM	0.90	0.54	0.51	0.52	0.73
LR	0.90	0.51	0.56	0.53	0.75

**Table 3:** Results of different methods with an under-sampling technique.

Methods	Accuracy	Precision	Recall	F-Score	AUC
Without balance	0.90	0.58	0.41	0.48	0.69
Random under-sampling	0.74	0.28	0.78	0.41	0.76
TomekLinks [40]	0.90	0.59	0.41	0.48	0.69
NearMiss [41]	0.37	0.15	0.95	0.26	0.62
CondensedNearestNeighbour [42]	0.85	0.39	0.58	0.47	0.73
OneSidedSelection [43]	0.90	0.59	0.41	0.48	0.69
NeighbourhoodCleaningRule [38]	0.90	0.56	0.51	0.53	0.72
EditedNearestNeighbours [39]	0.89	0.55	0.54	0.54	0.74
RepeatedEditedNearestNeighbours [40]	0.88	0.48	0.56	0.52	0.74
AllKNN [40]	0.89	0.51	0.55	0.53	0.74
InstanceHardnessThreshold [44]	0.85	0.40	0.63	0.49	0.75

**Table 4:** Results of different methods with an over-sampling-based method.

Methods	Accuracy	Precision	Recall	F-Score	AUC
Without balance	0.90	0.58	0.41	0.48	0.69
Random over-sampling	0.87	0.47	0.55	0.51	0.73
SMOTE [45]	0.88	0.47	0.54	0.50	0.73
Borderline-SMOTE type 1 [46]	0.88	0.48	0.55	0.51	0.73
Borderline-SMOTE type 2 [46]	0.87	0.46	0.60	0.52	0.76
Support Vectors SMOTE [36]	0.89	0.53	0.50	0.51	0.72
ADASYN [47]	0.89	0.53	0.49	0.51	0.72
SMOTE + Tomek [48]	0.88	0.47	0.55	0.51	0.73
SMOTE + ENN [49]	0.88	0.47	0.54	0.50	0.73

**Table 5:** Results of the over-sampling-based approach.

Methods	Accuracy	Precision	Recall	F-Score	AUC
Balanced Bagging DT [50]	0.81	0.32	0.67	0.43	0.75
Balanced RandomForest [51]	0.73	0.26	0.76	0.39	0.75
EasyEnsemble [52]	0.74	0.26	0.76	0.38	0.75
RUSBoost [53]	0.74	0.26	0.76	0.38	0.75

**Table 6:** Results of removing 1000 most important features.

Method	Accuracy	Precision	Recall	F-Score	AUC
LR with DB	0.90	0.51	0.56	0.53	0.75
LR with DB and FS	0.90	0.51	0.62	0.56	0.78
1000 best features removed	0.84	0.29	0.30	0.29	0.60

**Table 7:** Results of the different types of features selected.

Feature Name	Selected	Original	Category (%)	Overall (%)
text	332	5000	6.6	4.90
synset vector	471	2000	23.5	6.90
sentiments	3	5	60.0	0.04
cluster vector	123	981	12.5	1.80
structural features	2	3	66.7	0.03
adrlexicon	2	2	100.0	0.03
topics	1	1	100.0	0.01
topic vector	65	500	13.0	0.95
goodbad	1	4	25.0	0.01

**Table 8:** Results by deep learning methods.

Methods	Accuracy	Precision	Recall	F-Score	AUC
CNN [54]	0.88	0.47	0.50	0.48	0.71
CRNN [54]	0.85	0.38	0.53	0.44	0.71
RCNN [54]	0.89	0.50	0.44	0.46	0.69
BERT with BCE	0.90	0.56	0.50	0.53	0.85
BERT with MSE	0.91	0.62	0.45	0.52	0.86
BERT with fixed weights-1	0.90	0.58	0.49	0.51	0.82
BERT with fixed weights-2	0.91	0.53	0.55	0.53	0.83
BERT with BAW	0.90	0.56	0.53	0.55	0.87

## Data Availability

The data used to support the findings of this study are included in the article.

## References

[1] Benton A., Ungar L., Hill S., Hennessy S., Mao J., Chung A., Leonard C.E., Holmes J.H. (2011). Identifying Potential Adverse Effects Using the Web: A New Approach to Medical Hypothesis Generation. J. Biomed. Inform..

[2] Mao J.J., Chung A., Benton A., Hill S., Ungar L., Leonard C.E., Hennessy S., Holmes J.H. (2013). Online Discussion of Drug Side Effects and Discontinuation among Breast Cancer Survivors. Pharmacoepidemiol. Drug Saf..

[3] Freifeld C.C., Brownstein J.S., Menone C.M., Bao W.J., Filice R., Kass-Hout T., Dasgupta N. (2014). Digital Drug Safety Surveillance: Monitoring Pharmaceutical Products in Twitter. Drug Saf..

[4] Liu J., Zhao S., Zhang X. (2016). An Ensemble Method for Extracting Adverse Drug Events from Social Media. Artif. Intell. Med..

[5] Pierce C.E., Bouri K., Pamer C., Proestel S., Rodriguez H.W., Le H.V., Freifeld C.C., Brownstein J.S., Walderhaug M., Edwards I.R. (2017). Evaluation of Facebook and Twitter Monitoring to Detect Safety Signals for Medical Products: An Analysis of Recent Fda Safety Alerts. Drug Saf..

[6] Sarker A., Gonzalez G. (2015). Portable Automatic Text Classification for Adverse Drug Reaction Detection via Multi-corpus Training. J. Biomed. Inform..

[7] Liu J., Zhao S., Wang G. (2018). SSEL-ADE: A Semi-supervised Ensemble Learning Framework for Extracting Adverse Drug Events from Social Media. Artif. Intell. Med..

[8] Dai H.J., Wang C.K. (2019). Classifying Adverse Drug Reactions from Imbalanced Twitter Data. Int. J. Med. Inform..

[9] Sarker A., Ginn R., Nikfarjam A., O’Connor K., Smith K., Jayaraman S., Upadhaya T., Gonzalez G. (2015). Utilizing Social Media Data for Pharmacovigilance: A Review. J. Biomed. Inform..

[10] Pappa D., Stergioulas L.K. (2019). Harnessing Social Media Data for Pharmacovigilance: A Review of Current State of the Art, Challenges and Future Directions. Int. J. Data Sci.

[11] Vaswani A., Shazeer N., Parmar N., Uszkoreit J., Jones L., Gomez A.N., Kaiser L., Polosukhin I. (2017). Attention Is All You Need. Adv. Neural Inf. Process Syst..

[12] BERT: Pre-Training of Deep Bidirectional Transformers for Language Understanding. https://aclanthology.org/N19-1423.

[13] Predicting Adverse Drug Events from Personal Health Messages. https://www.ncbi.nlm.nih.gov/pmc/articlws/PMC3243174/.

[14] Quantifying Self-Reported Adverse Drug Events on Twitter: Signal and Topic Analysis. https://dl.acm.org/doi/pdf/10.1145/2930971.2930977.

[15] Sampathkumar H., Chen X.W., Luo B. (2014). Mining Adverse Drug Reactions From Online Healthcare Forums Using Hidden Markov Model. BMC Medical Inform. Decis. Mak..

[16] Nikfarjam A., Sarker A., O’Connor K., Ginn R. (2015). Pharmacovigilance from Social Media: Mining Adverse Drug Reaction Mentions Using Sequence Labeling with Word Embedding Cluster Features. J. Am. Med. Inform. Assoc..

[17] Mining Adverse Drug Reaction Signals from Social Media: Going beyond Extraction. https://www.researchgate.net/publication/280446645.

[18] Shabani-Mashcool S., Marashi S.-A., Gharaghani S. (2020). NDDSA: A Network- and- domain-based Method for Predicting Drug-side Effect Associations. Inf. Process Manag..

[19] Ding Y., Tang J., Guo F. (2019). Identification of Drug-Side Effect Association via Multiple Information Integration with Centered Kernel Alignment. Neurocomputing.

[20] One Size Does Not Fit All: An Ensemble Approach towards Information Extraction from Adverse Drug Event Narratives. https://researchr.org/publication/biostec-2019hi.

[21] Kim Y., Meystre S.M. (2020). Ensemble Method–Based Extraction of Medication and Related Information from Clinical Texts. J. Am. Med. Inform. Assoc..

[22] El-allaly E., Sarrouti M., En-Nahnahi N., El Alaoui S.O. (2021). MTTLADE: A Multi-Task Transfer Learning-Based Method for Adverse Drug Events Extraction. Inf. Process Manag..

[23] Magge A., Sarker A., Nikfarjam A., Gonzalez-Hernandez G. (2019). Deep Learning for Pharmacovigilance: Recurrent Neural Network Architectures for Labeling Adverse Drug Reactions in Twitter posts. J. Am. Med. Inform. Assoc..

[24] A Deep Learning Approach to Extracting Adverse Drug Reactions. https://computer.org/csdl/proceedings/aiccsa/2019/1ifhrWozXb2.

[25] Cocos A., Fiks A.G., Masino A.J. (2017). Deep Learning for Pharmacovigilance: Recurrent Neural Network Architectures for Labeling Adverse Drug Reactions in Twitter Posts. J. Am. Med. Inform. Assoc..

[26] Wang C.S., Lin P.J., Cheng C.L., Tai S.H. (2019). Detecting Potential Adverse Drug Reactions Using a Deep Neural Network Model. J. Medical Internet Res..

[27] El-allaly E.D., Sarrouti M., En-Nahnahi N., El Alaoui S.O. (2019). A LSTM-Based Method with Attention Mechanism for Adverse Drug Reaction Sentences Detection. Proceedings of the International Conference on Advanced Intelligent Systems for Sustainable Development.

[28] BioReddit: Word Embeddings for User-Generated Biomedical NLP. https://aclanthology.org/D19-6205/.

[29] KFU NLP Team at SMM4H 2019 Tasks: Want to Extract Adverse Drugs Reactions from Tweets? BERT to the Rescue. https://www.aclanthology.org/W19-3207/.

[30] Lee J., Yoon W., Kim S., Kim D., Kim S., So C.H., Kang J. (2019). Biobert: A Pre-Trained Biomedical Language Representation Model for Biomedical Text Mining. Bioinformatics.

[31] Fan B., Fan W., Smith C. (2020). Adverse Drug Event Detection and Extraction from Open Data: A Deep Learning Approach. Inf. Process Manag..

[32] ADR Classification. https://diego.asu.edu/Publications/ADRClassify.html.

[33] High-Dimensional Data Analysis: The Curses and Blessings of Dimensionality. https://citeseerx.ist.psu.edu/viewdoc/summary?doi=10.1.1.329.3391.

[34] Lemaître G., Nogueira F., Aridas C.K. (2017). Imbalanced-Learn: A Python Toolbox to Tackle the Curse of Imbalanced Datasets in Machine Learning. J. Mach. Learn. Res..

[35] The Machine Learning Tool Sciki-Learn. https://scikit-learn.org/.

[36] Borderline Over-Sampling for Imbalanced Data Classification. https://ousar.lib.okayama-u.ac.jp/en/19617.

[37] Janocha K., Czarnecki W.M. On Loss Functions for Deep Neural Networks in Classification. https://arxiv.org/abs/1702.05659.

[38] Laurikkala J. (2001). Improving Identification of Difficult Small Classes by Balancing Class Distribution. Proceedings of the Conference on Artificial Intelligence in Medicine in Europe.

[39] Wilson D.L. (1972). Asymptotic Properties of Nearest Neighbor Rules Using Edited Data. IEEE Trans. Syst. Man Cybern. Syst..

[40] Tomek I. (1976). An Experiment with the Edited Nearest-neighbor Rule. IEEE Trans. Syst. Man Cybern. Syst..

[41] KNN Approach to Unbalanced Data Distributions: A Case Study Involving Information Extraction. https://www.site.uottawa.ca/~nat/Workshop2003/jzhang.pdf.

[42] Hart P. (1968). The Condensed Nearest Neighbor Rule. IEEE Trans. on Information Theory.

[43] Kubat M., Matwin S. (1997). Addressing the Curse of Imbalanced Training Sets: One-sided Selection. Proceedings of the International Conference on Machine Learning.

[44] Smith M.R., Martinez T., Giraud-Carrier C. (2014). An Instance Level Analysis of Data Complexity. Mach. Learn..

[45] Chawla N.V., Bowyer K.W., Hall L.O., Kegelmeyer W.P. (2002). SMOTE: Synthetic Minority Over-Sampling Technique. J. Artif. Intell. Res..

[46] Han H., Wang W.Y., Mao B.H. (2005). Borderline-SMOTE: A New Over-sampling Method in Imbalanced Data Sets Learning. Proceedings of the International Conference on Intelligent Computing.

[47] He H., Bai Y., Garcia E.A., Li S. (2008). ADASYN: Adaptive Synthetic Sampling Approach for Imbalanced Learning. Proceedings of the IEEE International Joint Conference on Neural Networks.

[48] Balancing Training Data for Automated Annotation of Keywords: A Case Study. https://citeseerx.ist.psu.edu/viewdoc/download?doi=10.1.1.10.2192.

[49] Batista G.E., Prati R.C., Monard M.C. (2004). A Study of the Behavior of Several Methods for Balancing Machine Learning Training Data. ACM Trans. Knowl. Discov. Data.

[50] Louppe G., Geurts P. (2012). Ensembles on Random Patches. Proceedings of the Joint European Conference on Machine Learning and Knowledge Discovery in Databases.

[51] Using Random Forest to Learn Imbalanced Data. https://statistics.berkeley.edu/sites/default/files/tech-reports/666.pdf.

[52] Liu X.Y., Wu J., Zhou Z.H. (2008). Exploratory Undersampling for Class-imbalance Learning. IEEE Trans. Syst. Man Cybern. Syst..

[53] Seiffert C., Khoshgoftaar T.M., Van Hulse J., Napolitano A. (2009). RUSBoost: A Hybrid Approach to Alleviating Class Imbalance. IEEE Trans. Syst. Man Cybern. Syst..

[54] Adverse Drug Reaction Classification with Deep Neural Networks. https://aclanthology.org/C16-1084/.

